# Prognostic Value of Lymph Node Ratio in Cutaneous Melanoma: A Systematic Review

**DOI:** 10.7759/cureus.19117

**Published:** 2021-10-29

**Authors:** Jaffar Khan, Asad Ullah, Nathaniel Matolo, Abdul Waheed, Noor Nama, Nitasha Sharma, Kalyani Ballur, Lauren Gilstrap, Sohni G Singh, Intisar Ghleilib, Joseph White, Frederick D Cason

**Affiliations:** 1 Department of Pathology and Laboratory Medicine, Indiana University School of Medicine, Indianapolis, USA; 2 Pathology, Medical College of Georgia - Augusta University, Augusta, USA; 3 Surgical Oncology, San Joaquin General Hospital, French Camp, USA; 4 Surgery, San Joaquin General Hospital, French Camp, USA; 5 Obstetrics and Gynaecology, Bolan Medical College Complex Hospital Quetta, Quetta, PAK; 6 Pathology and Laboratory Medicine, Medical College of Georgia - Augusta University, Augusta, USA

**Keywords:** age group, overall survival, metastatic disease, lymph node ratio, prognosis, melanoma, cutaneous

## Abstract

The prognosis of cutaneous melanoma (CM) is based on the histological characteristics of the primary tumor, such as Breslow depth, ulceration, and mitotic rate. The lymph node ratio (LNR) is the ratio of the involved lymph nodes (LNs) divided by the total number of LNs removed during regional LN dissection. LNR is a prognostic factor for many solid tumors; however, controversies exist regarding CM. This study sought to analyze the role of LNR as a prognostic factor in CM.

An extensive literature search was conducted using PubMed, Google Scholar, Medline, and the Cochrane Central Registry of Controlled Trials from January 1966 to July 2015. The keywords included in the search were CM and inclusion of the ratio of positive to the total number of LNs as a prognostic factor. The outcomes analyzed included the number of patients with positive LNs, type of survival analysis, and results from the multivariate analysis.

A total of 11 studies involving 12,011 patients with positive LNs were evaluated. No previous randomized controlled trials, meta-analyses, or systematic reviews were identified in the Cochrane database on the prognostic value of LNR in CM. The primary electronic database search resulted in 333 full-text articles. The LN location examined was the cervical, axillary, and inguinal regions in all studies except for one that examined only the inguinal region. All studies except three studied the prognostic value of the LNR as a categorical variable rather than a continuous variable. LNR was categorized as A (≤0.1), B (0.11-0.25), and C (>0.25). All studies identified LNR as an independent predictor of overall survival (OS), disease-free survival (DFS), or disease-specific survival (DSS). The hazard ratio (HR) and confidence interval (CI) associated with either DFS or OS were available only in a few studies. Moreover, pooled HR for OS was 2.08 (95% CI: 1.48 2.92), for DFS was 1.364 (95% CI: 0.92-2.02), and for DSS was 1.643 (95% CI: 0.89-3.0).

The LNR provides superior prognostic stratification among patients with CM. Additional adequately powered prospective studies are needed to further define the role of LNR and be included in the staging system of CM and direct adjuvant therapy.

## Introduction and background

Cutaneous melanoma (CM) is the fifth most common cancer in the United States, with a 1.1% mortality rate among all cancer deaths [1,2]. In comparison to other cancer types, the incidence of CM is increasing every year [3,4]. The incidence of CM varies among different geographic regions due to various racial skin phenotypes and the degree of sun exposure. It primarily affects young to middle-aged individuals, with females in the younger age group and males usually after 50 years [5].

Similarly, ultraviolet (UV) radiation from the sun is an environmental risk factor, with sun exposure related to various skin diseases. Intense sun exposure is associated with a higher risk than chronic continuous exposure, which is associated with conditions such as actinic keratosis and non-melanoma skin cancers [6]. Furthermore, lymph node (LN) positivity is an important prognostic factor for CM [7]. The survival rate reduces to <10% in cases where melanoma spreads beyond the regional LNS [8]. Sentinel LN involvement in CM is the primary determinant of the staging and clinical management of these patients [9].

Robust advancements in oncology have introduced many chemotherapeutic agents that aim to increase the survival rate in patients with CM. Adjuvant immunotherapies directed against several antibodies, such as anti-cytotoxic T-lymphocyte-associated antigen 4 (anti-CTLA-4), anti-programmed cell death protein 1 (anti-PD-1), and BRAF/mitogen-activated protein kinase (MEK) inhibitors, have improved disease-free survival (DFS) [10]. Despite several studies on CM, no consensus has been established on using the lymph node ratio (LNR) as a prognostic factor and including it in staging cancer. This systematic review aimed to establish the role of LNR as a prognostic factor in patients with CM.

## Review

Methodology

Criteria for Literature Search

**Figure 1:**
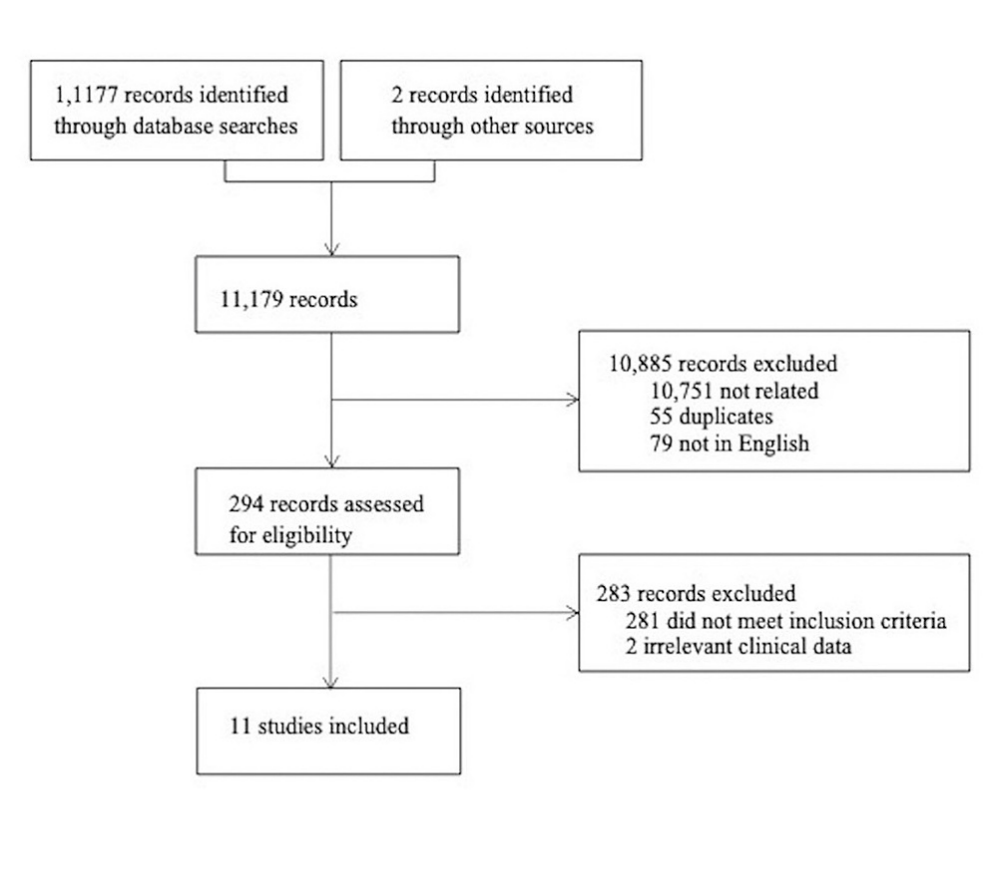
CONSORT diagram of the study selection process using the PRISMA protocol. CONSORT: Consolidated Standards of Reporting Trials; PRISMA: Preferred Reporting Items for Systematic Reviews and Meta-Analyses

A comprehensive literature search of all articles concerning CM and LNR was performed using PubMed, Google Scholar, Medline, and the Cochrane Central Registry of Controlled Trials from 1966 to 2015. Moreover, various national and international conference websites were also searched. All selected articles were published in English. Preferred Reporting Items for Systematic Reviews and Meta-Analyses (PRISMA) guidelines were followed (Figure 1). Keywords searched included “Melanoma,” “cutaneous,” “lymph node,” and “ratio.” No randomized control trials (RCTs) were found in the Cochrane Library during the literature search for the study period.

Data Extraction

The articles retrieved after the extensive literature search were evaluated for quality using an internal source. Three authors assessed the validity of these articles. If there was a conflict in selecting an article to be included in the study, a third author’s agreement and judgment were considered. The primary clinical outcomes were LN positivity in patients with CM, the type of survival analysis, and the results from multivariate analysis. The details of the methodology and quality are summarized in Tables 1-3.

**Table 1:** Demographic and methodological details of LNR studies in CM. DSS: disease-specific survival; DFS: disease-free survival; OS: overall survival; LNR: lymph node ratio; CM: cutaneous melanoma

Study	Patient age	Study period	Follow-up time	Outcome parameters
Sandro et al. [1]	Median 54 years	1992–2011	Median 54 months	DSS
Grotz et al. [2]	Median 54 years	1997–2010	Median 44 months	DFS, OS
Egger et al. [3]	Mean 47.6 years	1997–2003	Median 59 months	DFS, OS
Wevers et al. [4]	Median 58 years	2003–2011	Median 18 months	DFS, DSS
Berger et al. [5]	Mean 57.9 years	1993–2007	Median 26 months	OS
Van der Ploeg et al. [6]	Median 54 years	1991–2009	Median 20 months	DFS, OS
Mocellin et al. [7]	Median 56 years	1998–2006	Median 39 months	DSS
Spillane et al. [8]	Mean 57 years	1993–2006	Median 68 months	DFS, OS
Brown et al. [9]	Median 51 years	1997–2003	Median 68 months	DFS, OS
Xing et al. [10]	Mean 50 years	1988–2005	Median 39 months	DSS
Rossi et al. [11]	Mean 50 years	1990–2005	Mean 40 months	OS

**Table 2:** Methodological quality of the identified studies. LN: lymph node; SEER: Surveillance, Epidemiology, and End Results

Study	Year	Number of patients with positive LNs	Location examined	Study design
Sandro et al. [1]	2015	2,526	Cervical, axilla, inguinal	Retrospective multicenter database study
Grotz et al. [2]	2013	411	Cervical, axilla, inguinal	Retrospective multicenter cohort study
Egger et al. [3]	2013	345	Cervical, axilla, inguinal	Post hoc analysis
Wevers et al. [4]	2012	149	Cervical, axilla, inguinal	Prospective single-center study
Berger et al. [5]	2012	168	Cervical, axilla, iliac, inguinal	Retrospective single-center study
Van der Ploeg et al. [6]	2011	169	Iliac, inguinal	Prospective single-center study
Mocellin et al. [7]	2011	3,872	-	Retrospective SEER database study
Spillane et al [8]	2010	1,514	Cervical, axilla, inguinal	Prospective database study
Brown et al. [9]	2010	296	-	Prospective multi-institutional study
Xing et al. [10]	2009	2,348	Cervical, axilla, inguinal	Retrospective SEER database study
Rossi et al. [11]	2008	213	Cervical, axilla, iliac, obturator, inguinal	Prognostic single-institute study

**Table 3:** Summary of prognostic outcome data. LNR: lymph node ratio; MSS: melanoma-specific survival; AJCC: American Joint Committee on Cancer; TNM: Tumor, Node, Metastasis; LN: lymph node; TLND: therapeutic lymph node dissection; DSS: disease-specific survival; NSN: non-sentinel node; DFS: disease-free survival; OS: overall survival

Study	LNR categories	Univariate survival analysis	Multivariate survival analysis	Prognostic significance of LNR
Sandro et al. [1]	A (≤0.1), B (0.11–0.25), C (>0.25)	Significant	Significant	Significant predictor of MSS in AJCC N1a and AJCC N2a-positive LNs after SLNB
Grotz et al. [2]	<0.15, ≥0.15	Significant	Significant	Important prognostic factor in stage III melanoma but not independent over the current AJCC TNM staging system
Egger et al. [3]	A (≤0.1), B (0.10–0.25), C (>0.25)	Significant	Significant	Only sentinel LN-positive patients were considered. The prognostic value of LNR was inferior to alternative measures of LN disease
Wevers et al. [4]	Continuous	Significant	Not significant	Better prognosis after TLND for stage IIIB-C melanoma when LN metastasis is located in the neck compared to axillary and groin regions. Only patients with clinically positive LN were considered
Berger et al. [5]	A (≤0.1), B (0.11–0.25), C (>0.25)	Significant	Significant	The LNR model had a better fit for survival than AJCC N stage
Van der Ploeg et al. [6]	A (≤0.1), B (0.11–0.25), C (>0.25)	Significant	Significant	Inguinal LNR and groin LN metastasis were considered
Mocellin et al. [7]	Continuous value	NA	Significant	Independently predicted DSS, improving the prognostic accuracy of the TNM system. Good prognosis in patients with ≥10 excised LNs
Spillane et al. [8]	A (≤0.1), B (0.10–0.25), C (>0.25)	Significant	Not significant	Significant for AJCC N3 stage. LNR is an independent prognostic variable that can substage melanoma patients
Brown et al. [9]	Continuous value	Significant	Significant	NSN melanoma metastasis is an independent prognostic factor for DFS and OS. Only SLN-positive patients were considered
Xing et al. [10]	Neck: 0.07 Axilla: 0.13 Inguinal: 0.18	Significant	Significant	Significant prognostic factor for patients with head and neck or limb cutaneous melanoma
Rossi et al. [11]	A (≤0.1), B (0.11–0.25), C (>0.25)	Significant	Significant	Independent prognostic factor for melanoma patients with LN metastases

Statistical Analysis

All data were entered into an Excel sheet which was then imported into the Comparative Meta-Analysis Software version 3 (Biostat, Englewood, NJ). The hazard ratios (HRs) and associated confidence intervals (CIs) were calculated.

Results

Demographic Characteristics of the Studies

A total of 11 studies involving 12,011 patients with positive LNs were evaluated in this review. No previous RCTs, meta-analyses, or systematic reviews were identified in the Cochrane database regarding the prognostic value of LNR in patients with CM. The primary electronic database search resulted in 333 full-text articles.

Lymph Node Data

The LN location examined was the cervical, axillary, and inguinal in all studies except one that examined only the inguinal region. In all but one study, the prognostic value of the LNR was assessed in the presence of possible confounding covariates using Cox multivariate regression. All studies except three studied the prognostic value of the LNR as a categorical variable rather than a continuous variable. LNR was categorized as A (≤0.1), B (0.11-0.25), and C (>0.25).

Survival Data

**Figure 2:**
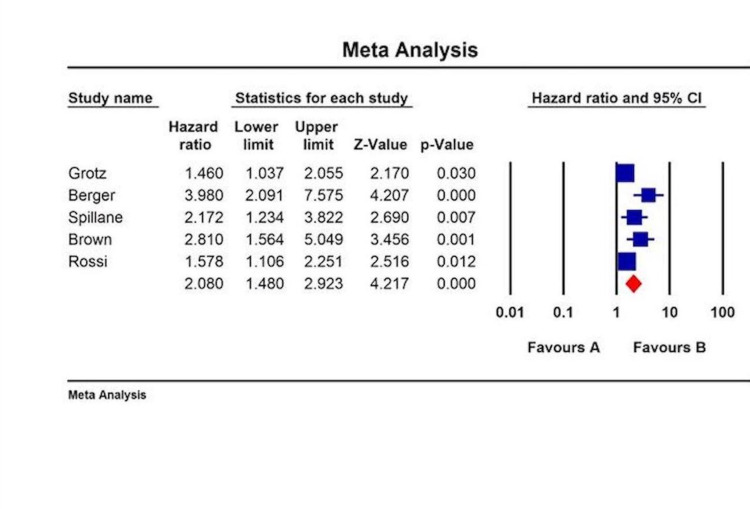
OS categorized by LNR in melanoma patients by multivariate analysis. OS: overall survival; LNR: lymph node ratio; CI: confidence interval

**Figure 3:**
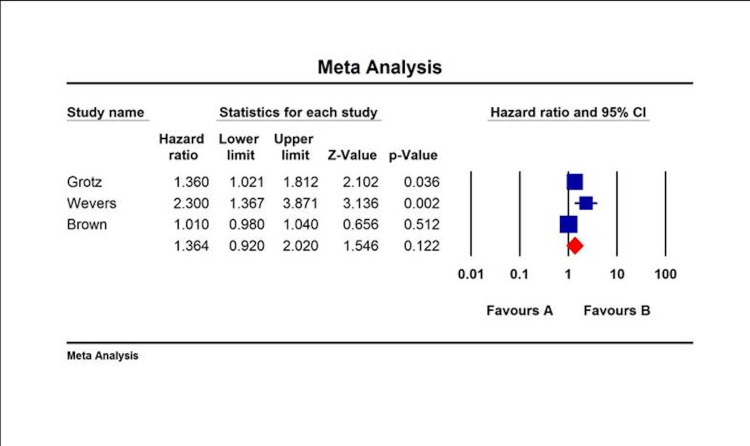
DFS categorized by LNR in melanoma patients by multivariate analysis. DFS: disease-free survival; LNR: lymph node ratio; CI: confidence interval

All included studies revealed that LNR was an independent predictor of overall survival (OS), disease-free survival (DFS), or disease-specific survival (DSS). The HR and CI associated with either DFS or OS were available only in a few studies. The collective HR for OS was 2.08 (95% CI: 1.48-2.92) (Figure 1), for DFS was 1.364 (95% CI: 0.92-2.02) (Figure 2), and for DSS was 1.643 (95% CI: 0.89-3.0) (Figure 3).

**Figure 4:**
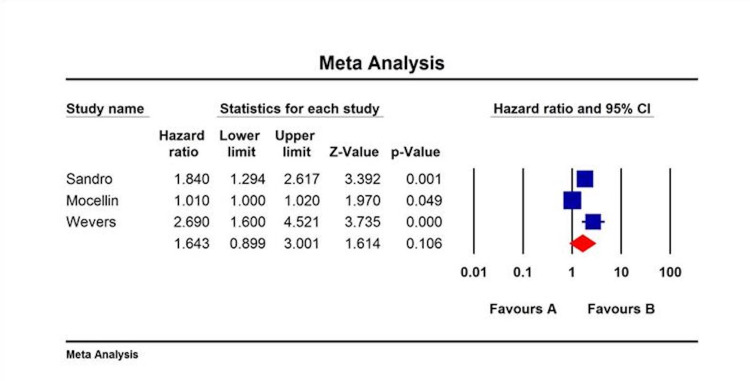
DSS categorized by LNR in melanoma patients by multivariate analysis. DSS: disease-specific survival; LNR: lymph node ratio; CI: confidence interval

Discussion

The prevalence of CM has increased over the past few decades. In 2018, the prevalence of CM in men and women was 663,784 and 631,102, respectively [11]. One study found that, from 2006 to 2015, the incidence increased in the United States, except in adolescents and young adults aged 10-19; this age group experienced a decrease in the incidence of CM [12]. The decrease in melanoma cases in this age group is likely attributed to public health efforts endorsing UV protection [12]. Based on data from 2015 to 2017, the overall risk of developing melanoma in the United States was 4.84% in men and 3.25% in women [13]. Although the prevalence of melanoma is lower than that of other skin cancers, accounting for less than 5% of cutaneous cancers, most skin cancer-related deaths can be attributed to melanoma [13].

Regarding the pathogenesis of CM, it primarily develops from abnormal melanocytes in the epidermal basal layers of the skin [14]. Typical melanocytes produce melanin which absorbs UV rays and protects against UV-induced DNA damage [14]. When DNA damage occurs and goes unrepaired due to genetic or environmental errors in DNA repair mechanisms, these cells can invade other cutaneous layers or spread to other areas of the body, such as LNs, the pulmonary system, the hepatic system, or the brain [14,15]. One study revealed that melanoma is most often diagnosed during a skin self-check or routine skin examination by a dermatology provider [16]. Abbasi et al. reported that melanoma presents as either a visual change in an existing nevus or a new nevus and that these changes are assessed with the “ABCDE” mnemonic outlining the common characteristics of CM, namely, asymmetry, border irregularity, color variegation, diameter >6 mm, and evolution in appearance [17].

Additionally, more advanced investigations play a crucial role in the diagnosis and management of CM. Genetic sequencing of melanoma specimens has been shown to help optimize treatment through targeted therapy [18]. Mutations in the mitogen-activated protein kinase (MAPK) cell signaling pathway are commonly observed in CM [19]. Interestingly, with most MAPK pathway mutations, the murine sarcoma viral oncogene homolog mutation, also known as BRAF mutation, is present in 40-60% of melanomas, and the neuroblastoma ras viral oncogene homolog mutation is found in 15-30% [19].

Utilizing genomic diagnostic techniques is crucial for developing treatment strategies for CM as specific mutations are known to be associated with advanced disease progression [20]. For example, compared to intact or wild-type BRAF melanomas, Ribas et al. found that mutant BRAF melanomas were more likely to metastasize to the brain [20]. Moreover, Hughdahl et al. found that BRAF mutant melanomas were associated with decreased survival in patients with stage IV melanoma compared to wild-type BRAF [21].

Although various therapies have evolved over the past few decades, the primary management of CM remains surgical resection [21]. In addition, the extent of the surgical margins is based on the thickness of the melanoma as the cells can grow beyond the existing border [16]. Wide excision is aimed at histologically negative margins to prevent recurrence [21]. If LNs are involved, lymphadenectomy along with wide excision may be beneficial. A randomized study evaluating the role of sentinel node biopsy on the outcomes of patients with melanoma by Morton et al. found that, among patients who had a positive sentinel node biopsy, those who underwent LN dissection had an overall five-year survival rate of 72.3% compared to a five-year survival rate of 52.4% in patients who had a delayed LN dissection [22]. These findings support complete lymphadenectomy following nodal metastases to improve survival in patients with melanoma. Adjuvant radiotherapy is indicated for positive margins, lymphatic invasion, recurrent melanoma, primary sites in the head or neck, and desmoplastic neurotropic growth [22]. In a study assessing the recurrence and survival of patients who underwent surgical excision and hypofractionated radiation therapy, Stevens et al. found that 11% of patients who underwent radiation therapy had recurrence at a median duration of six months, as well as a five-year survival rate of 0% and 46% for patients with and without recurrence, respectively [23].

Personalized therapy focusing on targeting signaling pathways has received significant attention. Vemurafenib was the first Food and Drug Administration (FDA)-approved agent in 2011 to target BRAF V600E; however, resistance and relapse developed with reactivation of the MAPK pathway [22-24]. The side effects of vemurafenib include photosensitivity and squamous cell carcinoma [22,23]. In 2013, dabrafenib was approved by the FDA for unresectable or metastatic BRAF-mutated melanoma [20-23]. Fever and cutaneous complications are side effects associated with dabrafenib [19-23]. Combination therapy with BRAF and MAPK/ERK kinase (MEK) inhibitors, such as trametinib and cobimetinib, is the recommended treatment for BRAF-mutated melanoma [19-23]. In a trial comparing survival in patients with unresectable late-stage BRAF V600E or V600K-mutated melanoma receiving either combination therapy of dabrafenib and trametinib and those receiving dabrafenib and placebo, Long et al. concluded that combination therapy reduced the risk of disease progression by 25% and improved response [24].

Regulatory T-cells in patients with melanoma may cause CD8+ T-cells to respond weakly to melanoma antigens, creating an opportunity to use immunotherapeutic agents that allow T-cells to avoid regulatory T-cell checkpoints [24]. Ipilimumab, an antibody that blocks CTLA-4, is effective in patients with wild-type BRAF [22-25]. Hodi et al. evaluated the survival of patients with previously treated metastatic melanoma and found that the median survival was 10.0 months for patients receiving ipilimumab and glycoprotein 100 peptide vaccine. In comparison, the median survival was 6.4 months for patients receiving the glycoprotein 100 peptide vaccine alone [25]. Nivolumab and pembrolizumab, antibodies that block PD-1, have higher response rates and improved progression-free and overall survival than ipilimumab [25-27].

Inferior clinical outcomes have been observed in men compared to women with melanoma. In a study comparing melanoma outcomes in men and women, Behbahani et al. reported that men were diagnosed at 61 years of age, while women were diagnosed earlier at a median age of 55 years [28]. In the same study, women had a mean OS of 11.1 years and a 10-year survival rate of 73.1%, while men had a mean OS of 9.6 years and a 10-year survival rate of 58.7%. Even after adjusting for confounding variables, women had better survival rates and prognoses. The better outcomes in women may be attributed to the earlier age of diagnosis, the lower stage of melanoma and LN metastasis at the time of diagnosis, and milder skin sensitivity to UV light than men [29].

Limitation

Like most studies involving literature review, our study has a few limitations. As most databases do not carry all the published literature, there might be a possibility that we might have missed some studies even after an extensive literature review. We validated the quality of the studies internally using a three-contributor technique and did not validate the external quality of the studies. Furthermore, we did not examine the socioeconomic impact of the patients in detail.

## Conclusions

Despite the study limitations, we believe that sufficient information was gathered for a generalized understanding of the topic. LNR provides superior prognostic stratification among patients with CM. Additional adequately powered prospective studies are needed to further define the role of LNR and be included in the staging system of CM and direct adjuvant therapy.
